# Use of nanoparticle concentration as a tool to understand the structural properties of colloids

**DOI:** 10.1038/s41598-017-18573-7

**Published:** 2018-01-17

**Authors:** Lígia Nunes de Morais Ribeiro, Verônica Muniz Couto, Leonardo Fernandes Fraceto, Eneida de Paula

**Affiliations:** 1 Department of Biochemistry and Tissue Biology, Institute of Biology, University of Campinas (UNICAMP), Campinas, São Paulo Brazil; 2 São Paulo State University (UNESP), Institute of Science and Technology of Sorocaba, Laboratory of Environmental Nanotechnology, Sorocaba, São Paulo Brazil

## Abstract

Elucidation of the structural properties of colloids is paramount for a successful formulation. However, the intrinsic dynamism of colloidal systems makes their characterization a difficult task and, in particular, there is a lack of physicochemical techniques that can be correlated to their biological performance. Nanoparticle tracking analysis (NTA) allows measurements of size distribution and nanoparticle concentration in real time. Its analysis over time also enables the early detection of physical instability in the systems not assessed by subtle changes in size distribution. Nanoparticle concentration is a parameter with the potential to bridge the gap between *in vitro* characterization and biological performance of colloids, and therefore should be monitored in stability studies of formulations. To demonstrate this, we have followed two systems: extruded liposomes exposed to increasing CHCl_3_ concentrations, and solid lipid nanoparticles prepared with decreasing amounts of poloxamer 188. NTA and dynamic light scattering (DLS) were used to monitor changes in nanoparticle number and size, and to estimate the number of lipid components per particle. The results revealed a strong negative correlation between particle size (determined by DLS) and concentration (assessed by NTA) in diluted samples, which should be adopted to monitor nanocolloidal stability, especially in drug delivery.

## Introduction

Colloids are defined as complex systems composed of solid particles dispersed in a liquid, governed by a high internal kinetic energy^1^. They are widely studied in biological, food and environmental fields, and Ostwald said ‘all life processes take place in a colloidal system’^2^. Characterization of these dynamic systems is the first and essential step for innovative nanocolloid development. However, there is still a lack of methods that can help to predict the *in vivo* performance of nanostructured colloids^3^.

There are many processes that can affect the physicochemical stability of colloidal systems, such as agglutination, sedimentation and Ostwald ripening. In the case of drug delivery systems, for instance, the resulting heterogeneous size distribution and larger particle size^1^ prevent their application. Therefore, accelerated or long-term study of physicochemical stability must be performed for those formulations^4^. In general, parameters such as particle size and distribution are followed over time by particle-tracking approaches based on the Brownian motion principle^5^, such as photocorrelation spectroscopy/dynamic light scattering (DLS), laser diffraction analysis and nanoparticle tracking analysis (NTA). DLS is the gold standard method applied in stability monitoring of formulations. Its measurement considers the intensity of light scattered by spherical particles, which prevents its application for highly polydisperse^6^ and non-spherical colloidal systems.

NTA is a novel, number-based tracking approach^7^ that provides unique information of the dispersed nanoparticle concentration of a known volume in real time^8^. Determination of the concentration does not depend on the intensity of the light scattered by the particles^9^ and, therefore, it can also be applied for the characterization of non-spherical particles such as carbon nanotubes, clays or fluorescent aggregates. Its use and implications have guided different biological studies, such as cell viability, cyto- and nanotoxicity assays and *in vivo* efficacy studies in pharmaceutical and environmental fields^10–14^, as well as in clinical treatment (e.g. monitoring and diagnosis in chronic lymphocytic leukaemia)^15^. Although promising, the use of nanoparticle concentration is still scarce in the literature, probably due to the indispensable operator training for the so-called ‘visual validation’ of NTA determination^8^. Until 2011, only two works had employed the number of nanoparticles as a parameter to evaluate nanotoxicity in ecotoxicity/environmental studies, against 18 others that compared DLS and NTA in the characterization of particle size and distribution^9^.

In 2016, when NTA was 10 years old, the International Standard Particle Tracking Analysis Guide (ISO 19430) was launched, providing specifications for the measurement of particle size distribution and concentration in nanosystem characterization using NTA. The guide preconizes the use of number-based particle size distribution and direct results interpretation, and specifies quality technical parameters, among others^16^. Nanoparticle concentration seems to be the bridge over the current abyss between *in vitro* characterization data and *in vivo* performance of nanosystems.

In this study, we firstly compared DLS (standard) and NTA (novel) in the evaluation of particle size and polydispersity of extruded liposomes composed of hydrogenated soy phosphatidylcholine: cholesterol (HSPC:Chol, 2: 1 mol%). The accuracy of the NTA method for laboratorial formulations was confirmed for this (two lipid) heterogeneous formulation. Then, taking into account that size distribution is a good indicator of instability for colloidal systems, we checked whether changes in this parameter would directly affect the number of nanoparticles in the system. For that, we intentionally destabilized unilamellar liposomes (composed of dipalmitoyl phosphatidylcholine, DPPC) and solid lipid nanoparticles (SLN, composed of cetyl palmitate and poloxamer 188) to evaluate the correlation between particle size (measured by DLS) and nanoparticle concentration (NTA). In this context, we show here the advantages of using NTA to investigate the stability of colloidal systems, focusing on those designed for the delivery of active molecules (drug delivery systems).

## Results

### Following the decrease in size during extrusion of liposomes

**Figure 1:**
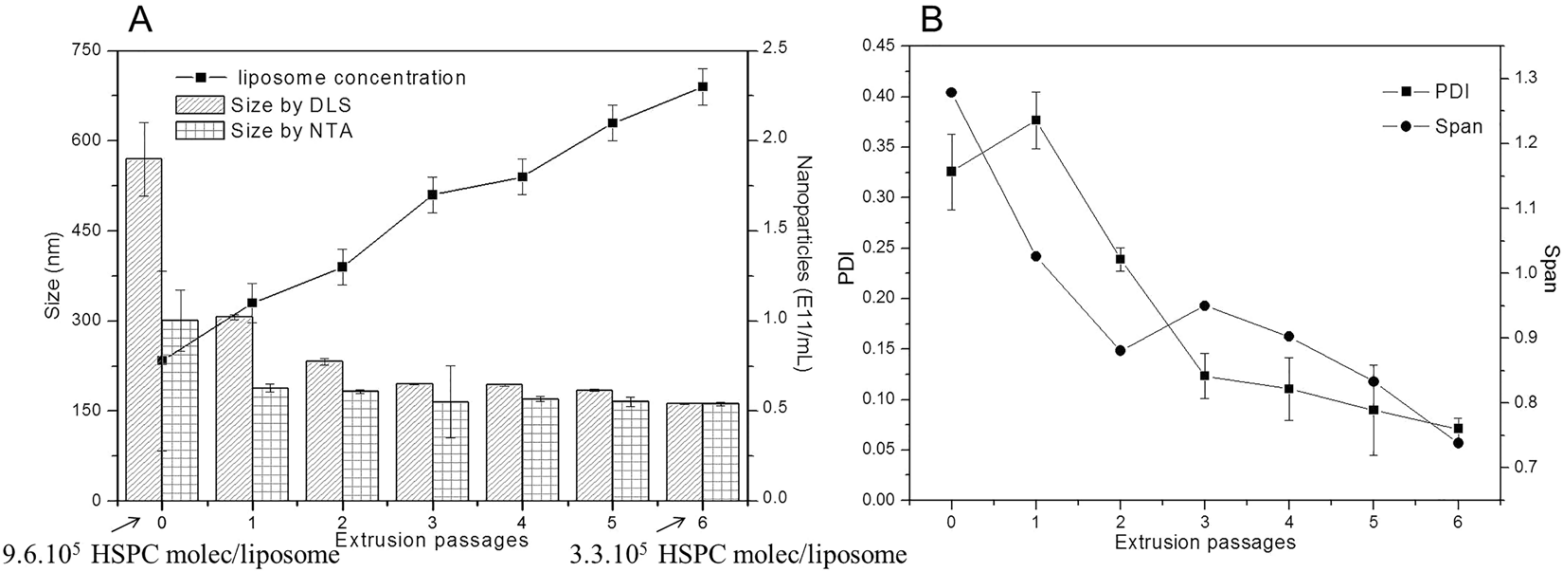
HSPC:Chol (2: 1 mol%) liposomes. Relationship between particle size, measured by DLS (undiluted samples) or NTA (200× dilution), and nanoparticle concentration (**A**), and polydispersity and Span indices (**B**) as a function of the number of extrusion passages. The correlation coefficients between the particle size values measured by DLS and NTA, and PDI *vs*. Span values through the extrusion cycle were: r = 0.98 and 0.75, respectively. At the bottom of plot A, the number of HSPC molecules/liposome for the initial (non-extruded) and six-times extruded vesicles are given. [HSPC:Chol] = 0.125 mM.

The particle size distribution of HSPC:Chol (2:1 mol%) liposomes was determined using DLS and NTA techniques (Fig. 1). Data were recorded after each extrusion passage (from 0 to 6). The particle size of non-extruded liposomes (0 passages) was 569 ± 61.2 and 301 ± 51.1 nm according to DLS and NTA, respectively. For liposomes extruded once (1), the sizes decreased to 306 ± 4.0 nm by DLS and 188 ± 6.8 nm by NTA (Fig. 1A), with polydispersity index (PDI) and Span values around 0.4 and 1.3 (Fig. 1B), respectively. For liposomes submitted to the complete extrusion cycle (6 times), the particle sizes observed were 162.5 ± 1.4 nm by DLS and 162.4 ± 2.8 nm by NTA (Fig. 1A), with PDI <0.150 and Span <1 (Fig. 1B), respectively.

On the other hand, the nanoparticle concentration varied from 7.8 × 10^10^ particles/mL for non-extruded liposomes to 2.3 × 10^11^ particles/mL (Fig. 1A) after the complete (6) extrusion cycle. Accordingly, the estimated number of HSPC molecules per particle – taken from the lipid concentration (mol/mL) divided by the number of nanoparticles/mL – decreased from 9.6 × 10^5^ (non-extruded) to 3.3 × 10^5^ after six extrusion passages (Fig. 1A).

### Destabilization of nanostructured colloidal systems

Two different colloidal systems were prepared, and their dispersed nanoparticles were destabilized to form larger ones. Transmission electron microscopy (TEM) was carried out to characterize the great morphological changes in the colloidal systems after destabilization. During the destabilization process, particle size and concentration (assessed by DLS and NTA, respectively) were followed, and the correlation coefficient (r) between them was registered.

#### DPPC liposomes

**Figure 2:**
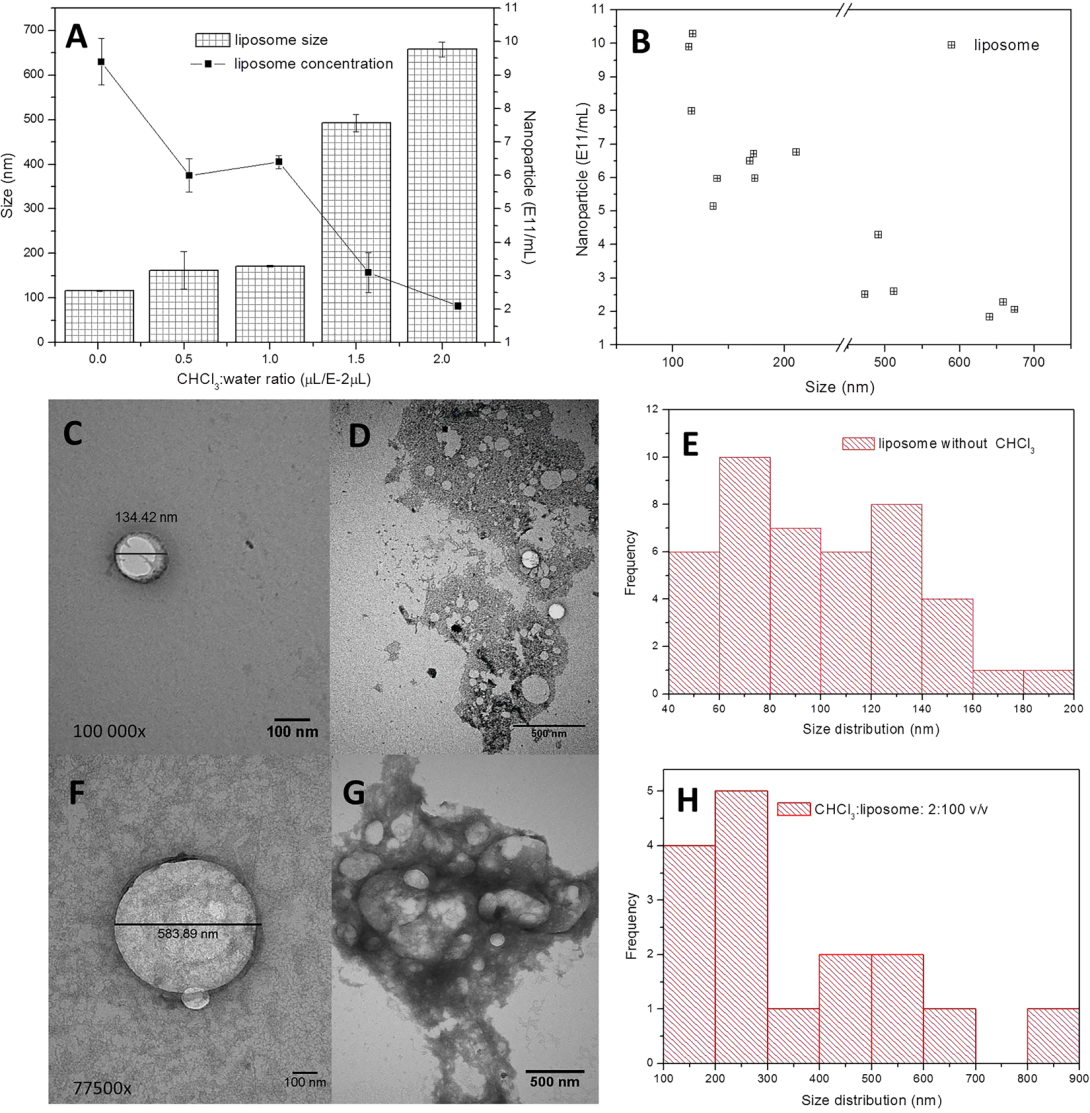
Destabilization of DPPC liposomes by chloroform. (**A**) Changes in particle size (measured by DLS) and nanoparticle concentration (assessed by NTA); (**B**) correlation between particle size and concentration (r = −0.92). TEM images of intact liposomes (**C**,**D**) and liposomes treated with CHCl_3_ (2: 100 v/v) (**F**,**G**), and their corresponding particle size distribution profile (**E**,**H**) calculated with ImageJ software. Magnifications and scale bars are displayed per sample.

First, liposomes composed of DPPC (0.125 mM) were extruded six times through 100 nm polycarbonate membranes. The resulting unilamellar vesicles were then destabilized with increasing chloroform (CHCl_3_) concentrations. The addition of small aliquots of CHCl_3_ to the extruded liposomes increased the particle size measured by DLS, from 116 ± 1.8 nm (CHCl_3_-free) to 658 ± 17 nm (Fig. 2A), in the presence of CHCl_3_ (2: 100 v/v).

The increase in particle size was confirmed by TEM (Fig. 2C,D,F and G), in which ImageJ software allowed quantification of the particle size distribution (Fig. 2E and H). Figures 2C and D show the untreated liposomes, and Figures 2F and G show liposomes treated with CHCl_3_ (2: 100 v/v).

Inversely, the nanoparticle concentration decreased with increasing CHCl_3_ concentrations, from 9 ± 0.7 × 10^11^ particles/mL (without chloroform) to 2 ± 0.1 × 10^11^ particles/mL (CHCl_3_ 2: 100 v/v), as measured by NTA (Fig. 2A), accompanied by an increase in the number of DPPC molecules per liposome, from 8.0 × 10^4^ to 35.7 × 10^4^ (Table 1). A strong negative correlation (r = −0.92) between particle size (DLS) and nanoparticle concentration (NTA) values was detected, as shown in Fig. 2B.

**Table 1:** Structural effects in liposomes destabilized by CHCl_3_ addition, evaluated in terms of size (DLS) and concentration (NTA) of particles.

CHCl_3_ (molecules/mL)	Size (nm)	Liposome concentration (particles/mL)	DPPC per liposome (molecules/particle)
0	116.5 ± 1.8	9.4 ± 0.7 × 10^11^	8.0 × 10^4^
7.2 × 10^16^	171.8 ± 2.3	6.4 ± 0.2 × 10^11^	11.7 × 10^4^
11.4 × 10^16^	491.9 ± 19.4	3.1 ± 0.6 × 10^11^	24.2 × 10^4^
15.0 × 10^16^	657.6 ± 17.0	2.1 ± 0.1 × 10^11^	35.7 × 10^4^

The number of DPPC molecules per vesicle was calculated from the number of particles and DPPC concentration (mol/L) in the formulation.

#### Solid lipid nanoparticles

**Figure 3:**
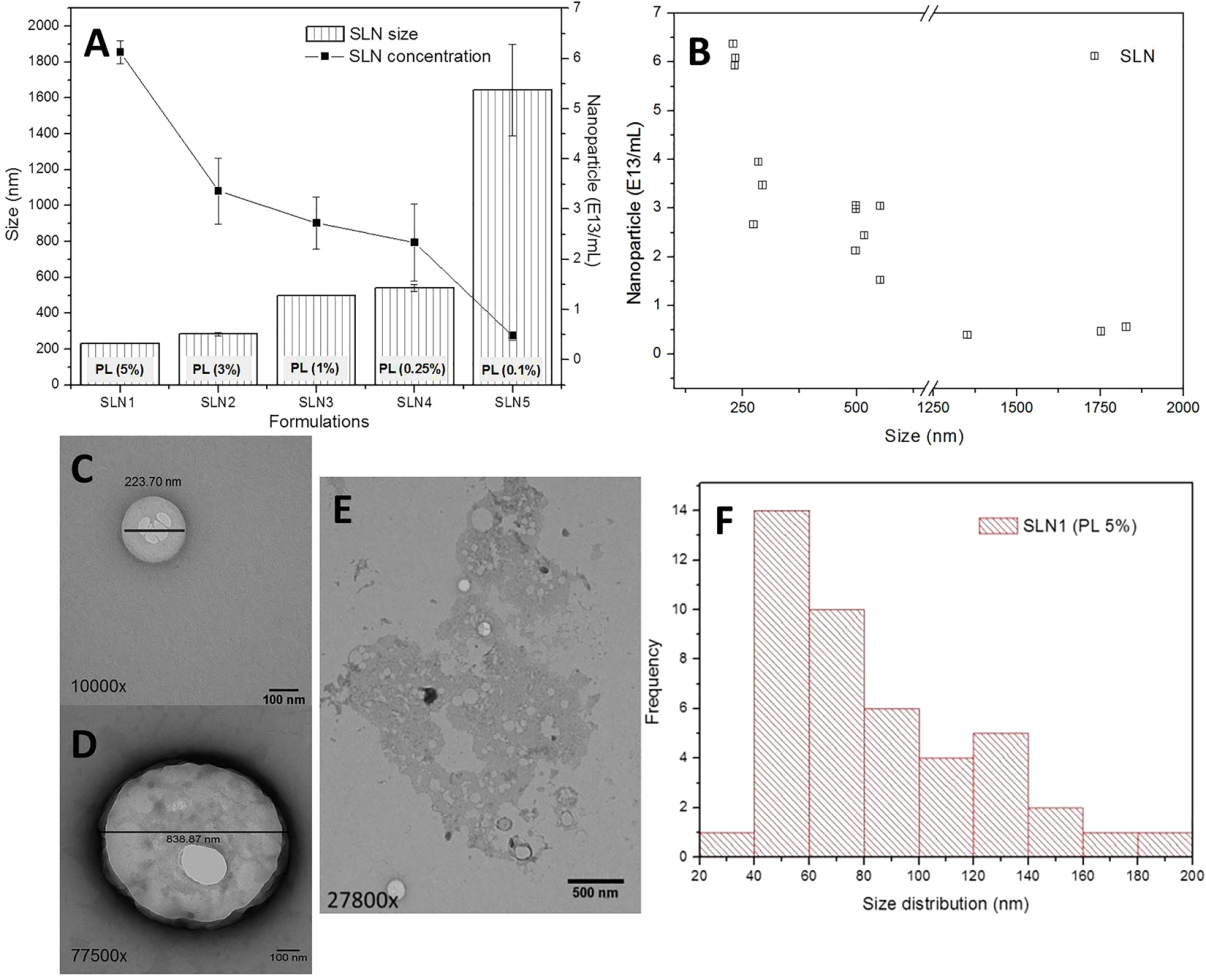
Destabilization of SLN formulations. (**A**) Changes in particle size (measured by DLS) and nanoparticle concentration (assessed by NTA) in SLN composed of 10% cetyl palmitate and decreasing concentrations (5 to 0.1%) of poloxamer 188; (**B**) correlation between particle size and concentration (r = −0.81). (**C**,**E**) TEM images of SLN1, prepared with 5% PL, and its corresponding particle size distribution profile (**F**), calculated with ImageJ software; (**D**) TEM image of SLN5, prepared with 0.1% PL, for which it was not possible to obtain the distribution size profile (see text). Magnification and scale bars are displayed per sample.

Five SLN formulations with the same lipid concentration (cetyl palmitate, CP = 10%) and decreasing concentrations of the non-ionic surfactant poloxamer 188 (PL = 5% to 0.1%) were prepared and analysed by DLS, NTA (Fig. 3A), and TEM (Fig. 3C–F). A reduction in PL concentration induced changes in particle size – which ranged from 232 to 1644 nm – accompanied by a decrease in nanoparticle concentration – from 6.0 × 10^13^ to 0.4 × 10^13^ particles/mL. The inverse correlation between particle size (DLS) and nanoparticle concentration (NTA) was detected (r = −0.81, Fig. 3B). Accordingly, the number of CP molecules per SLN increased from 2.0 × 10^6^ to 2.6 × 10^7^, as shown in Table 2.

**Table 2:** Structural effects in SLN prepared with different poloxamer 188 ratios, evaluated in terms of size (DLS) and concentration (NTA) of particles.

Formulation	PL concentration (%)	Size (nm)	SLN concentration (particles/mL)	CP per SLN (molecules/particle)
SLN1	5	232.1 ± 2.7	61.3 ± 0.2 × 10^12^	2.0 × 10^6^
SLN2	3	284.2 ± 9.9	33.6 ± 0.6 × 10^12^	3.7 × 10^6^
SLN3	1	499.1 ± 0.4	27.2 ± 0.5 × 10^12^	4.6 × 10^6^
SLN4	0.25	540.9 ± 20.5	23.3 ± 0.8 × 10^12^	5.4 × 10^6^
SLN5	0.1	1644.3 ± 255.9	4.8 ± 0.1 × 10^12^	2.6 × 10^7^

The number of cetyl palmitate (CP) molecules per SLN was calculated from the number of nanoparticles and CP concentration in the formulation (mol/L).

The variation in particle size observed for SLN1 and SLN5 was also demonstrated by TEM (Fig. 3C–E). However, quantification of the particle size distribution using ImageJ software was only possible for SLN1 (Fig. 3F) since the polydispersity of SLN5 curbed its micrographic analysis.

## Discussion

A considerable number of studies have compared the DLS and NTA methods, showing the accuracy, advantages and drawbacks of each technique, and considering their peculiarities. However, all those studies have employed standard nanoparticles, such as polystyrene beads^8,17,18^. Such ideal systems do not reproduce the size distribution of formulations developed in research laboratories, which have a less homogeneous size distribution. Hence, to compare the DLS and NTA methods, we prepared different and more realistic lipid-based colloidal systems: liposomes and SLN.

DLS is the gold standard method for characterizing colloidal systems, despite its poor accuracy for highly polydisperse samples and non-spherical particles^8^. This explains the differences in particle size of liposomes with 0 or 1 extrusion passages, measured by DLS and NTA. The particle-by-particle counting approach adopted by the NTA method provides robust characterization of heterogeneous particle size distribution, since – differently to DLS – changes in the intensity of light scattered by large and small particles do not affect determination of the hydrodynamic diameter by NTA^9^. This is particularly useful for the characterization of a colloidal system formed by a complex mixture of submicron and nanometric particles^17^, such as biomimetic systems (liposomes) and membrane fractions (e.g. exosomes, buddings, detergent-resistant membranes)^7^.

### Size vs. number of particles during extrusion of liposomes

As expected, the particle size, PDI, and Span values of liposomes decreased as a function of the number of extrusion cycles, measured by both methods. At the end of the extrusion cycle, the liposome diameters determined by these two techniques were very similar and strongly positively correlated. Accordingly, the PDI (DLS) and Span (NTA) values were lower than 0.2 and 1, respectively, characterizing a monodisperse distribution^19,20^. Both techniques were analytical for characterization, but NTA has the advantage of using highly diluted samples (ca. 200 times higher than DLS). These results demonstrated the accuracy of NTA in the determination of particle size and nanoparticle concentration in colloidal systems.

Changes in liposome concentration (particles/mL) during the extrusion cycle were also monitored (Fig. 1A) with NTA. As expected, the extrusion process led to a higher number of small particles originated from the larger multilamellar ones^21^. From non-extruded liposomes to liposomes submitted to the complete (6) extrusion cycle, a directly proportional increase in the number of nanoparticles was registered. Yet the decrease in vesicle size was accompanied by enhancement in the number of liposome particles in suspension, suggesting that these parameters are related and should be explored in more depth.

### Estimation of the number of lipid molecules per liposome particle

**Figure 4:**
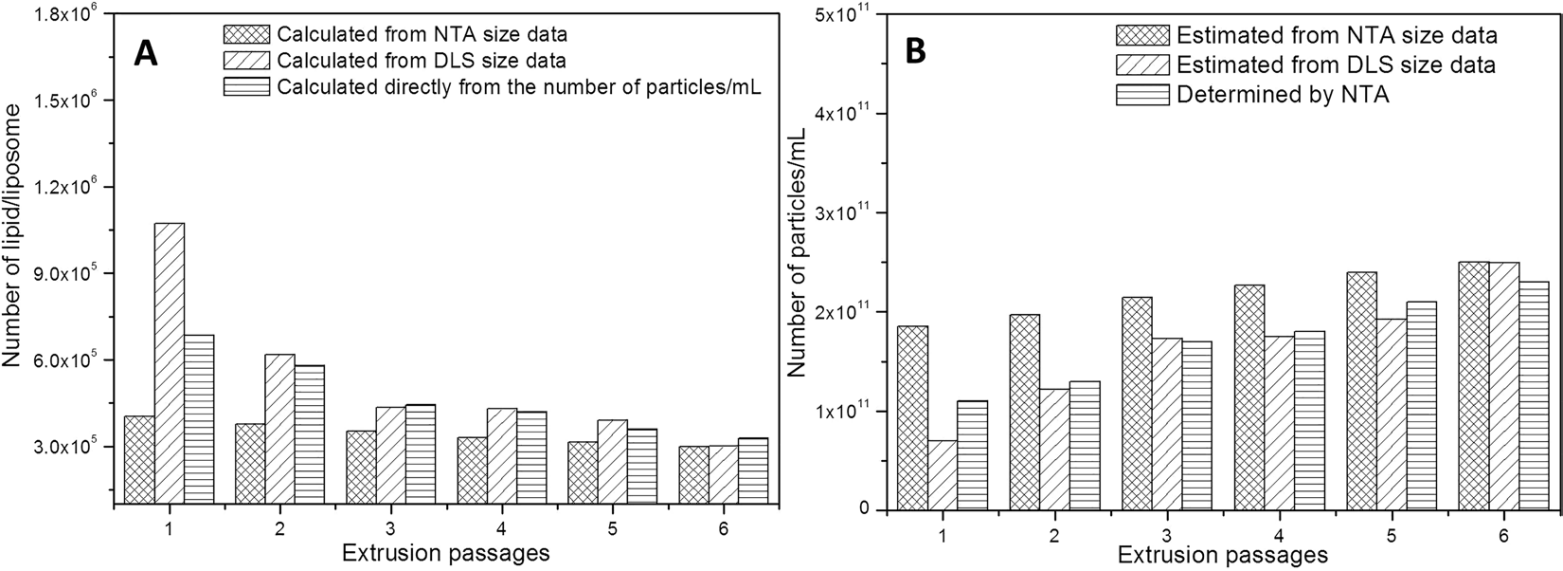
Theoretically *vs*. experimentally determined number of lipid molecules/liposome (**A**) and number of particles/mL (**B**) during the extrusion process. The number of HSPC molecules that could be loaded per liposome was estimated from the surface area of HSPC:Chol liposomes (considering the average size of the vesicles determined by DLS or NTA) divided by the area occupied by the lipid polar head. The number of HSPC molecules was also taken from the number of particles – determined by NTA – divided by the number of HSPC molecules in 0.125 mM suspension (7.5 × 10^16^ molecules).

During extrusion, the number of lipid molecules per liposome decreased, as exemplified for HSPC molecules (Fig. 1A). The number of HSPC/liposome, as shown in Table 1, was taken directly from the number of lipid molecules in the 0.125 mM liposomal suspension (7.5 × 10^16^) divided by the number of particles determined by NTA. Using an alternative method, as shown in Fig. 4A, we estimated the number of lipid molecules that could be loaded per liposome of a given size, and the values agree very well with those determined from NTA data. To estimate the number of lipids/vesicle, we first calculated the surface (sphere) area of the liposomes from their average sizes (measured during the extrusion process, either by DLS or NTA). Twice the surface area (equivalent to the internal and external monolayers of the liposomes) was then divided by the area occupied by each phosphatidylcholine molecule (55 Å^2^)^22^ to give the number of lipids/vesicle.

Also, by dividing the number of lipid molecules in the 0.125 mM liposomal suspension (7.5 × 10^16^) by the theoretical number of lipids/vesicle (estimated as indicated above – Fig. 4A), we could predict the number of particles/mL – according to DLS and NTA average size – as shown in Fig. 4B. As the extrusion process proceeds towards a monodisperse system, the predicted and determined numbers become closer and closer, converging at 2 × 10^11^ particles/mL. Once more, the good agreement found between the calculated nanoparticle concentration and that determined by NTA confirms the certainty and applicability of this technique.

### Destabilization of nanostructured colloidal systems

The most common events affecting the physicochemical stability of nanostructured colloids are coalescence, agglutination, and the Ostwald ripening. These processes result in larger and heterogeneous-sized nanoparticles^1^, preventing their application. In an attempt to mimic such processes, two different colloidal systems were prepared, and their dispersed nanoparticles were destabilized to form larger ones.

Liposomes composed of DPPC (0.125 mM) were destabilized upon addition of chloroform (CHCl_3_). The destabilizing effect of CHCl_3_ on lipid bilayers occurs by partial solubilization of the lipid’s acyl chains^23^, and has been broadly explored over decades^24^. The addition of CHCl_3_ aliquots provided a relevant increase of particle size, measured by DLS and confirmed by TEM. The absolute size measured by TEM differed to that measured by DLS because of the well-known limitations of this technique, such as shrinkage and deformation of nanoparticles during the drying process^25^ in sample preparation. Nevertheless, the micrographics confirmed the formation of larger liposomes following the CHCl_3_ treatment. Inversely, and as expected, the nanoparticle concentration decreased with increased CHCl_3_ concentration, confirming liposome destabilization.

In another set of experiments, a binary, and therefore more complex, lipid-based system of SLN was prepared. Five formulations with the same concentration of lipid (CP = 10%) and different amounts of the non-ionic surfactant PL (5 to 0.1%) were prepared. PL is known to stabilize lipid nanoparticles, physically preventing their agglutination^26^. Thus, at low PL concentrations, SLN stability was not maintained, contributing to nanoparticle aggregation (which prevented quantitative analysis by TEM). As expected, a lack of PL was accompanied by a drastic increase in particle size and number of lipids/particle, and by a decrease in nanoparticle concentration in SLN formulations (Fig. 3 and Table 2).

Altogether, destabilization of the nanostructured colloidal formulations was confirmed by all methods evaluated. The effects of destabilization were similar in both the liposome and SLN systems: an increase/decrease in nanoparticle size and concentration, respectively. The correlation coefficients between these parameters were determined for liposomes (r = −0.92, Fig. 2) and SLN (r = −0.81, Fig. 3). The correlation was higher for liposomes than SLN, probably due to the complex structural molecular organization of the latter. Considering the potential application of nanoparticle concentration in *in vitro* characterization and biological and clinical studies, such achievements indicate that particle size and concentration are strictly (inversed) related in different pharmaceutical colloidal systems, and should be used to monitor the physicochemical stability of formulations.

It is worth mentioning that monitoring of nanoparticle concentration over time can also help early detection of system instability, such as in the case of nanoparticle degradation, evidenced by a decrease in the number of particles without a concomitant increase in nanoparticle size. So far, only a few studies have adopted evaluation of nanoparticle concentration in the physicochemical stability analysis of pharmaceutical^4,27,28^ and environmental^13^ formulations.

The number of nanoparticles can be expressed as the molar concentration of colloidal particles in the formulation^11^, and this allows the estimation of the number of excipient molecules that compose each individual particle^4^, two important parameters that represent a real advance in the structural elucidation of nanocolloids.

We have shown here, for a single component formulation (DPPC liposomes), that the number of lipid molecules per vesicle can be determined from the nanoparticle concentration data, considering the number of lipid molecules in suspension (Table 1). Following the addition of solvent, the resulting destabilized liposomes (of greater size and lower concentration) showed increasing numbers of DPPC molecules per nanoparticle, in comparison to the initial (stable) vesicles. Similar results were observed with SLN (Table 2), for which destabilization was caused by low ratios of surfactant.

Taking into account the importance of nanoparticle concentration in determining the biological effects of colloidal systems, we foresee that there should be an ideal range for the number of nanoparticles in suspension, and we will endeavour to identify the acceptable limits of nanoparticle concentration. In this study we have shown that NTA is a simple, cost-effective, and real-time method for evaluating nanoparticle concentration. Therefore, it is essential to include this parameter in physicochemical stability studies of nanostructured colloidal systems intended for multipurpose applications, as well as to guarantee the quality of nanocolloidal products.

## Conclusions

The simple premise that significant changes in particle size and distribution indicate instability of a colloidal system formed the basis of our hypothesis. We have shown relevant evidence to support the idea that the concentration of nanoparticles measured by NTA should be a parameter followed in determining the physicochemical stability of colloids. The strong negative correlation between particle size assessed by DLS and nanoparticle concentration demonstrated that both parameters serve to monitor colloidal instability. In addition, in the case of nanoparticle instability (erosion, disruption or degradation), which directly affects colloidal system performance, the phenomenon can be prematurely predicted by monitoring the nanoparticle concentration, a measure that is not currently adopted in physicochemical stability studies. This is especially relevant due to increasing interest in this parameter as analytical information in biological and clinical studies. The number of colloidal particles in suspension may be the key to crossing the abyss between *in vitro* data and *in vivo* studies, reinforcing the relevance of monitoring nanoparticle concentration over time.

## Materials and Methods

### Materials

HSPC:Chol (2: 1 mol%) and 1,2-dipalmitoyl-sn-glycero-3-phosphocholine (DPPC) were purchased from Avanti Polar Lipids (USA), poloxamer 188 (PL) was supplied by Sigma (USA), cetyl palmitate (CP) was donated by Dhaymers Química Fina (Brazil) and 100 nm polycarbonate membrane filters were provided by Milipore (USA). All other reagents used were of analytical grade.

### Colloidal system preparation

#### Liposomes

Two different liposomal formulations were prepared, at a final lipid concentration of 0.125 mM: i) vesicles of DPPC, which were employed in liposome destabilization tests; and ii) vesicles of mixed composition, prepared with HSPC:Chol (2: 1 mol%), which were used to evaluate the number of extrusions necessary to reach a stable formulation of monodisperse particle size distribution.

Both formulations were prepared from stock chloroform lipid solutions. After the proper volumes of lipids were pipetted, the solvent was removed under nitrogen flux to create a thin lipid film in a round-bottom flask. The film was left to dry in a vacuum for 2 h to ensure the removal of any solvent trace. After that, the thin lipid film was hydrated with deionized water and vortexed (5 min) to form multilamellar liposomes.

DPPC multilamellar vesicles were extruded six consecutive times through polycarbonate membranes (100 nm pores) in order to obtain unilamellar vesicles of homogeneous size distribution^29^; the particle size and concentration of the final formulation were measured by DLS and NTA, respectively.

HSPC:Chol multilamellar liposomes were also extruded six times through the (100 nm pore) polycarbonate membranes, but after each extrusion, the size distribution and nanoparticle concentration were evaluated by DLS and NTA.

#### Solid lipid nanoparticles

SLN were prepared by the emulsification-ultrasonication method^30^. Specifically, the solid lipid (10% CP) was heated to 10 °C above its melting point, under magnetic stirring. The aqueous phase was prepared with appropriate amounts of PL in water, and heated to the same temperature as the lipid phase (64 °C). This phase was then dropped into the oily phase, under high-speed agitation (10,000 rpm) using a Turrax blender (IKA Werke Staufen, Germany) for 2 min. The obtained microemulsion was sonicated (Vibra-Cell, Sonics & Materials Inc., Danbury, USA) at 500 W and 20 kHz, in alternate 30 s cycles (on and off) for 15 min, and immediately cooled to 25 °C.

### Induced destabilization of colloidal systems

#### DPPC liposomes

DPPC liposomes were used to evaluate size distribution and nanoparticle concentration during destabilization induced by solvent. Briefly, small aliquots of chloroform were added to 1 mL of the liposome suspension to a final ratio of 0.5: 100, 1: 100, 1.5: 100 or 2: 100 (v/v), and the sealed vials were stirred for 30 s on a vortex. After 1 h, destabilization was monitored through DLS (particle size) and NTA (nanoparticle concentration) measurements.

#### Solid lipid nanoparticles

Five different SLN formulations were prepared, with the same concentration of lipid (10% or 208 mmol/L CP) and decreasing surfactant concentrations: 5%, 3%, 1%, 0.25% and 0.1% PL (SLN1 to SLN5, respectively). All formulations were characterized in terms of particle size (DLS) and nanoparticle concentration (NTA).

### Colloidal system characterization

#### Dynamic light scattering

Particle size and polydispersity index (PDI) of liposomes and SLN were analysed via laser light scattering (DLS) using Zetasizer Nano ZS90 equipment (Malvern Instruments, Worcestershire, UK). Liposomal samples were used without dilution but SLN samples were diluted 1000 times. The results are given as mean plus standard deviation (n = 3).

#### Nanoparticle tracking analysis

Particle size, Span, and nanoparticle concentration were assessed by NTA, carried out with a NanoSight NS300 (Malvern Instruments, Worcestershire, UK) equipped with a sample chamber and a 532 nm laser. The colloidal systems were diluted 200 times and 50,000 times, for liposomes and SLN, respectively, with around 2000 tracks counted in each measure. The colloidal systems were injected into the sample chamber with sterile syringes until the liquid extended to the tip of the syringe; measurements were performed in triplicate at room temperature.

#### Transmission electron microscopy

Transmission electron microscopy (TEM) images of liposomes and SLN formulations were measured in a JEOL 1200 EXII microscope, operated at 80 kV. Uranyl acetate (2%) was added to the diluted samples; after that, aliquots were dropped into copper grids coated with a carbon film, and dried at room temperature. After drying, micrographs of the formulations were taken at different magnifications.

### Data availability

All data generated or analysed during this study are included in this manuscript.
